# Understanding subtype-selective allosteric modulation of GABA_A_ receptors

**DOI:** 10.1186/2050-6511-13-S1-A26

**Published:** 2012-09-17

**Authors:** Roshan Puthenkalam, Zdravko Varagić, Pantea Mirheydari, Werner Sieghart, Margot Ernst

**Affiliations:** 1 Department of Biochemistry and Molecular Biology, Center for Brain Research, Medical University of Vienna, 1090 Vienna, Austria

## Background

The γ-aminobutyric acid type A (GABA_A_) receptors are the major inhibitory neurotransmitter receptors of the central nervous system. Benzodiazepine (Bz)-site ligands bind at the α/γ interface and can enhance GABA-induced Cl^−^ currents. The efficacy of certain benzodiazepines strongly depends on the type of α(1,2,3,5) subunits in the receptors. Functionally selective compounds for α2/3 can be anxiolytic without having the side effect of sedation. The molecular basis for functional selectivity is investigated in this work.

## Methods

Two-electrode voltage-clamp electrophysiology recordings were performed in wild-type and mutated receptors expressed in *Xenopus laevis* oocytes. Modelling, docking and molecular dynamics simulation studies of α1γ2 and α3γ2-containing receptors were performed to understand Bz-ligand interaction with the different α subunits.

## Results

Electrophysiology recordings identified flumazenil as a null modulator in α1 and a weak plus modulator in α3-containing receptors. A sequence comparison between the α1 and α3 subunit revealed the residue R228 as unique for the α3 subunit among all α subunits. α3R228A-mutated receptors completely lost their ability to respond to flumazenil. This amino acid is part of the so-called loop C, a several-residues-spanning segment that forms part of the ligand-binding site with a highly variable sequence. The functionally α3-selective ligand flumazenil was docked into the α/γ interface. The flumazenil-bound state in the α1 subtype has already been studied previously [1] and was used for comparison. Our results indicate that the binding mode of flumazenil in α1 and α3-containing receptors is very similar.

## Conclusions

The models made in this study show improved properties in certain variable segments that could not be resolved in the previously published models [1]. For understanding the role of α3R228, more models and docking computations have to be made on the basis of these improvements to explore possible conformations.
